# Comparison of Toxic Metal Distribution Characteristics and Health Risk between Cultured and Wild Fish Captured from Honghu City, China

**DOI:** 10.3390/ijerph15020334

**Published:** 2018-02-14

**Authors:** Jingdong Zhang, Liyun Zhu, Fei Li, Chaoyang Liu, Zhenzhen Qiu, Minsi Xiao, Ying Cai

**Affiliations:** 1 Research Center for Environment and Health, Zhongnan University of Economics and Law, Wuhan 430073, China; jdzhang@zuel.edu.cn (J.Z.); zhuliyun@zuel.edu.cn (L.Z.); lcy@zuel.edu.cn (C.L.); zzqiu@zuel.edu.cn (Z.Q.); msxiao@zuel.edu.cn (M.X.); 1993cy@zuel.edu.cn (Y.C.); 2 School of Information and Safety Engineering, Zhongnan University of Economics and Law, Wuhan 430073, China

**Keywords:** heavy metals, Honghu city, aquaculture production, target hazard quotient, carcinogenic risk, estimated weekly intake

## Abstract

Honghu Lake, which listed in the “Ramsar Convention”, is the seventh largest freshwater lake in China and is regarded as one of the biggest freshwater product output areas in China. The toxic element distribution in cultured and wild fish and the corresponding health risks through fish consumption from Honghu area were investigated. The mean concentration in the muscle of cultured and wild fish (*Carassius auratus* and *Ctenopharyngodon idellus*) decreased in the order: Zn (18.94) > Cu (0.8489) > Cr (0.2840) > Pb (0.2052) and Zn (16.30) > Cr (1.947) > Cu (0.4166) > Pb (0.0525) > Cd (0.0060) (mean; mg/kg, wet weight). Scales (Multi factor pollution index (MPI) = 3.342) and the liver (MPI = 1.276) were regarded as the main accumulation tissues for cultured fish, and the bladder (MPI = 0.640) and intestine (MPI = 0.477) were regarded as the main accumulation tissues for wild fish. There were no obvious health risks associated with the consumption of cultured and wild fish based on the calculated results of the target hazard quotient (THQ), carcinogenic risk (CR), and estimated weekly intake (EWI). Pb and Cr were recognized as the major health risk contributors for inhabitants through wild and cultured fish consumption. Cultured fish had a greater health risk than wild fish based on the calculation results of THQ and CR. Muscle consumption resulted in more health risks than mixed edible tissues for cultured fish, but for wild fish, the conclusion was the opposite. Mixed fish (cultured:wild = 1:1) muscle consumption had relatively lower risks than the consumption of cultured or wild fish muscle separately. Consuming no more than 465 g/day (wet wt) of cultured fish muscle, 68 g/day (wet wt) of wild fish muscle, 452 g/day (wet wt) of mixed cultured fish edible tissues or 186 g/day (wet wt) of mixed wild fish edible tissues from the Honghu area can assure human health.

## 1. Introduction

Based on aquaculture statistical data from the U.N.’s Food and Agricultural Organization (FAO, Rome, Italy), China is one of the most important contributors to global fishery production, with a total amount of 62,575 thousand tons, accounting for 37.42% of the global amount (167,228 thousand tons) in 2014 [1,2]. Freshwater aquaculture production is an important component of fisheries production, and the output value of freshwater aquaculture was 581,318 million yuan (approximately 89,606 million dollars), which accounted for 48.4% of the total fishery output value (approximately 185,016 million dollars) in 2016. Hence, the safety of freshwater aquaculture production from China affects the health of humans across the world [3].

Water used in aquaculture generally comes from surrounding river diversion, natural precipitation or well-water [4]. Recently, with agricultural non-point source pollution worsening and fishermen’s unscientific management, polluted water from livestock farms, farmland, and even chemical product factories has entered aquaculture through polluted river water [5]. Previous research on pollutants of freshwater fish mostly focused on antibiotics [6,7] and pesticides [8,9]. However, heavy metals and metalloids are also common pollutants in water or other environmental media and have attracted considerable attention due to their persistence, recalcitrance, bioaccumulation and potential toxicity, especially in some developing countries like China, Pakistan and India [10,11,12,13]. Heavy metals and metalloids that exceed standard concentrations will disturb the normal life activities of fish and even cause poisoning or death [14,15,16]. Heavy metals and metalloids can easily accumulate in fish through two main routes: a. through absorption by gills or ionic exchange of dissolved contaminants in water through biological membranes; b. through ingestion of contaminants in food or sediment particles [17,18]. Residual heavy metals and metalloids in fish will be amplified and enriched up the food chain, posing a serious threat to human health and the ecological environment. Thus, it is necessary to investigate heavy metals and metalloid distribution in cultured fish and analyze the corresponding health risks to humans.

Honghu city was recognized as the largest freshwater aquaculture production area in 2012 by the China aquatic products circulation and processing association. The freshwater aquaculture industry is an important part of the local economy. In 2016, the output value of fisheries in Honghu area reached 7249 million yuan, accounting for 57% of the total output value of local agriculture (12,712 million yuan). The yield of freshwater aquaculture production reached 485 thousand ton, and the freshwater aquaculture area covered 58.5 thousand hectares [19]. Recent research revealed that the surface water in Honghu Lake has been gradually polluted by heavy metals and metalloids due to anthropogenic activities [5,20,21], especially with respect to aquaculture activity [22]. Fish are popular in local inhabitants’ regular diet, not only for fish muscle, but also for some other edible fish tissues like the skin, bladder and liver, which have numerous nutrients and a pleasant taste sensation [23,24,25]. However, previous research on cultured fish consumption risk for human health mainly focused on fish muscle consumption [26,27,28]. Thus, it is necessary to investigate and compare the corresponding human health risk associated with fish muscle consumption and mixed edible fish tissues consumption. In order to analyze the factors affecting the heavy metal distribution in cultured fish, 2 popular fish species, wild *Carassius auratus* (abbreviated as Crucian carp) and *Ctenopharyngodon idellus* (abbreviated as Grass carp), which have been proved to have relatively higher trace element accumulation abilities in fish studies and some related studies [29,30], captured from Honghu Lake, were selected to make comparisons of toxic metal distributions and health risk assessments.

Overall, the specific objectives of this study are: (1) to investigate the trace element distributions in muscle and other tissues of cultured fish from local Honghu fishponds; (2) to compare the trace element distribution differences in muscles and tissues of cultured and wild fish; (3) to assess human health risks through cultured and wild fish consumption, to provide optimized fish consumption suggestions for local inhabitants; and (4) to analyze the fish daily consumption limit for local inhabitants with respect to cultured fish muscle, wild fish muscle, mixed cultured fish edible tissues and mixed wild fish edible tissues.

## 2. Materials and Methods

### 2.1. Study Area

Honghu city (E 113–114°, N 29–30°) is located in the middle of Hubei province. Honghu Lake (E 113°16–27′, N 29°46–55′, which covers about 348.3 km^2^ and has a length of 23.4 km from east to west and a width of 20.8 km from north to south), is the seventh largest freshwater lake in China and the largest nature wetland reserve in Hubei province. The climate of Honghu city belongs to subtropical monsoon; the annual mean precipitation is 1061–1331 mm, and the annual mean air temperature is 16.6 °C. The sampling fish farms are sporadically distributed around Honghu Lake, and the specific location of each fish farm is marked in Figure S1. The water temperature in Honghu Lake ranges from 27 °C–29 °C, and the mean pH value is 7.59 [31].

### 2.2. Sample Collection and Preparation

Cultured fish were bought from local fish farms. Wild fish were captured from Honghu Lake by local fishermen. All fish samples were carefully preserved in polyethylene sealing bags, and both fish length and weight were recorded and are presented in Tables S1 and S2. Samples were cryopreserved at −20 °C and transported to the laboratory as soon as possible. Essential information, i.e., the collected cultured and wild fish species and amounts, is presented in Tables S1 and S2.

All fish samples were carefully cleaned by ultra pure water and dissected into 7 parts (bladder, gill, intestine, liver, muscle, scale and skin) using stainless steel tools. Each part was gently dried using disposable filter paper and homogenized by a meat masher. All of the homogenized parts of fish samples were carefully preserved in small polyethylene bottles with category labels and transferred to a refrigerator at a temperature of −20 °C.

### 2.3. Sample Digestion and Analysis

The digestion procedures carefully followed the individual trace element detection methods from the national food safety standards [32,33,34,35,36,37]. Approximately 0.5 g of fish tissue was weighted into a digestion vessel containing 8 mL nitric acid (65%) and 2 mL hydrogen peroxide (30%). Vessels with mixed solutions were closed and allowed to stand for 20–30 min at room temperature and were then transferred to a microwave digestion system with a designated heated programming. After digestion, all solutions were placed into small porcelain mugs on an electric plate containing a base solution (0.2% diluent nitric acid), followed by heating at 120 °C until only 2–3 mL of the digestion solution remained to reduce the residual acid. Then, the solutions were diluted into 10 mL colorimetric tubes for storage and further detection by the base solution (0.2% diluent nitric acid). Cu, Zn, Cr, Cd, Pb were detected by Atomic Absorption Spectroscopy (AAS ZEEnit 700P, Jena, Germany) and As was detected by Atomic Fluorescence Spectrometry (AFS-9730, Haiguang, China) under appropriate analytical conditions. Instrument Limits of Detection (LODs, mg/kg) were 0.001 for Cd and As, 0.002 for Cr, 0.01 for Pb, 0.02 for Cu, and 0.1 for Zn.

All regents used, including nitric acid (65%) and hydrogen peroxide (30%), were of ultra-pure grade (Shanghai Sinopharm Group Chemical Reagent Limited Company, Shanghai, China). All experimental vessels for sample storage, digestion and detection were immersed overnight in nitric acid (20–30%) solution, rinsed in ultrapure water and dried in a clean laboratory oven. Quality assurance and quality control were carried out with parallel tests, blank tests and recovery tests [38,39,40]. Blank tests accompanied every batch of sample processing. A standard curve was drawn when the correlation coefficient was higher than 0.999 for all sample detection. The detection results were reliable when relative deviations of parallel sample analysis were below 10%, and the recovery rate ranged from 95% to 105%.

The single factor pollution index (abbreviated as P_i_) method is generally used to evaluate the pollution level of a single heavy metal [41,42]. The calculation equation is as follows:

(1) $$P_{i}=\frac{C_{i}}{S_{i}}$$

where C_i_ is the measured mean concentration of a single heavy metal (mg/kg, wet wt); S_i_ is the standard limit concentration for a single heavy metal (mg/kg, wet wt).

The multi factor pollution index (also called metal pollution index, and abbreviated as MPI) method is generally used to evaluate the total pollution level of multiple heavy metals [42,43]. The calculation equation is as follows:

(2) $$\mathrm{MPI}=\sqrt[{n}]{C_{1}\times C_{2}\times \ldots \times C_{n}}$$

where C_n_ is the measured mean concentration of the nth heavy metal (mg/kg, wet wt).

### 2.4. Health Risk Assessment

Target hazard quotient (abbreviated as THQ) is a kind of assessment method to evaluate the possible non-carcinogenic health risks due to chemical pollutant intake and was established by the United States Environmental Protection Agency [43]. This method assumes the intake dose is equal to the absorption dose, and cooking has no effect on the pollutants [44]. A THQ value less than 1 means there will be no obvious risk in consuming the studied fish sample, i.e., the exposure level of the study element is less than the reference dose. The calculation equation is listed as follows:

(3) $$\mathrm{THQ}=\frac{E_{F}\times E_{D}\times F_{\mathrm{IR}}\times C}{R_{\mathrm{FD}}\times W_{\mathrm{AB}}\times T_{A}}\times 10^{-3}$$

where E_F_ is the population exposure frequency (350 day/year); E_D_ is the exposure time (30 year); F_IR_ is the food daily consumption (54.33 g/day) [45]; C is the heavy metal concentration in food (mg/kg); R_FD_ is the reference oral dose (mg/kg/day); W_AB_ is the population average weight (61.6 kg) [46]; T_A_ is the non-carcinogenic average exposure time (365 day/year × E_D_) [47].

Carcinogenic risk (abbreviated as CR) is a kind of assessment method to evaluate the possible carcinogenic health risks due to chemical pollutant intake, such as As and Pb, and was also established by the United States Environmental Protection Agency [43]. The acceptable carcinogen risk level ranges from 10^−4^ (risk of developing cancer over a human lifetime is 1 in 10,000) to 10^−6^ (risk of developing cancer over a human lifetime is 1 in 1,000,000). The calculation equation is listed as follows:

(4) $$\mathrm{CR}=\frac{E_{F}\times E_{D}\times F_{\mathrm{IR}}\times C\times \mathrm{CSFO}}{W_{\mathrm{AB}}\times T_{A}}\times 10^{-3}$$

where E_F_ is the population exposure frequency (day/year); E_D_ is the exposure time (year); F_IR_ is the food daily consumption (g/day); C is the heavy metal concentration in food (mg/kg); CSFO is the oral carcinogenic slope factor from the Integrated Risk Information System database (mg/kg/day)^−1^ [48]; W_AB_ is the population average weight (kg); T_A_ is the non-carcinogenic average exposure time (day).

Estimated weekly intake (abbreviated as EWI) is employed to calculate the weekly trace element intake from food and was established by the World Health Organization (WHO) [49] and the United Nations Food and Agriculture Organization (FAO) [50]. Provisional tolerable weekly intake (abbreviated as PTWI) refers to the tentatively accepted weekly trace element intake. When the calculated value of EWI is lower than PTWI, it means there is no significant health risk for exposed population through food consumption. The calculation equation is listed as follows:

(5) $$\mathrm{EWI}=\frac{F_{\mathrm{IR}}\times C\times 7}{W_{\mathrm{AB}}}$$

where F_IR_ is the food daily consumption (g/day); C is the heavy metal concentration in food (mg/kg); W_AB_ is the population average weight (kg).

${\mathrm{FIR}}_{\lim}$ is the maximum allowable fish daily consumption for adults to assure their health. ${\mathrm{FIR}}_{\lim\left(\mathrm{THQ}\right)}$ is the maximum allowable fish daily consumption, which is calculated by the equation of THQ, ${\mathrm{FIR}}_{\lim\left(\mathrm{CR}\right)}$ is calculated by the equation of CR, and ${\mathrm{FIR}}_{\lim\left(\mathrm{EWI}\right)}$ is calculated by the equation of EWI.

(6) $${\mathrm{FIR}}_{\lim(\mathrm{THQ})}=\frac{R_{\mathrm{FD}}\times W_{\mathrm{AB}}\times T_{A}\times 1}{E_{F}\times E_{D}\times C}\times 10^{3}$$

where R_FD_ is the reference oral dose (mg/kg/day); W_AB_ is the population average weight (kg); T_A_ is the non-carcinogenic average exposure time (day); 1 is the limitation of THQ, THQ > 1 means the corresponding health risk is significant; E_F_ is the population exposure frequency (day); E_D_ is the exposure time (year); C is the heavy metal concentration in food (mg/kg).

(7) $${\mathrm{FIR}}_{\lim(\mathrm{CR})}=\frac{W_{\mathrm{AB}}\times T_{A}\times 10^{-4}}{E_{F}\times E_{D}\times C\times \mathrm{CSFO}}\times 10^{3}$$

where W_AB_ is the population average weight (kg); T_A_ is the non-carcinogenic average exposure time (day); 10^−4^ is the limitation of CR, CR > 10^−4^ means the corresponding health risk is unacceptable; E_F_ is the population exposure frequency (day/year); E_D_ is the exposure time (year); C is the heavy metal concentration in food (mg/kg); CSFO is the oral carcinogenic slope factor from the Integrated Risk Information System database (mg/kg/day)^−1^.

(8) $${\mathrm{FIR}}_{\lim(\mathrm{EWI})}=\frac{W_{\mathrm{AB}}\times \mathrm{PTWI}}{C\times 7}$$

where W_AB_ is the population average weight (kg); PTWI is the allowed heavy metal weekly intake, EWI > PTWI means the corresponding health risk is significant; C is the heavy metal concentration in food (mg/kg).

## 3. Results and Discussion

Table 1 shows the calculation results of the single factor pollution index (P_i_) and multi factor pollution index (MPI) of trace elements in cultured fish muscle. From the calculation results of MPI, the polluted levels of different species of cultured fish decreased in the following order: Grass carp > Catfish carp > Crucian carp > Carp.

Grass carp (regarded as cultured fish) has relatively high heavy metal accumulation ability. In addition, according to some published references [29,30], Crucian carp (regarded as wild fish) has relatively high heavy metal accumulation ability. Therefore, wild Grass carp and Crucian carp (captured from Honghu Lake) were selected as two typical fish species to make comparisons with cultured Grass carp and Crucian carp in trace element distribution and health risk assessment through consumption.

### 3.1. Trace Element Distributions in Muscle of Cultured and Wild Fish

The heavy metal (As, Cd, Cr, Cu, Pb and Zn) concentrations in muscle of cultured fish captured from fishponds around Honghu Lake and wild fish captured from Honghu Lake are listed in Table 2. The concentrations of As were lower than the limit of detection in both cultured and wild fish. Cd could not be detected in cultured fish muscles. The concentrations of Cu, Pb and Zn in muscles of cultured fish were relatively higher than those in wild fish; by contrast, the concentrations of Cr and Cd were higher in wild fish than in cultured fish.

Honghu Lake, located on the northern bank of the middle reach of the Yangtze River, is the seventh largest freshwater lake in China. Poyang Lake, located on the southern bank of the middle and lower reaches of the Yangtze River, is the largest freshwater Lake in China. Taihu Lake, located on the southern margin of the Yangtze River Delta, is the third largest freshwater Lake in China. Table 3 shows the trace element concentrations in wild fish muscles of those typical lakes that are linked to the Yangtze River. The concentrations of Cd, Cu and Pb in Honghu Lake wild fish muscles were close to the corresponding concentrations in Poyang Lake (Crucian carp and Grass carp); however, the concentrations of Cr and Zn were higher than those in Poyang Lake. The concentrations of all detected trace elements in Honghu Lake wild fish muscles except Cr were much lower than the corresponding concentrations in Taihu Lake (wild Crucian carp). The total concentrations of trace elements in the Yangtze River wild fish (Crucian carp and Grass carp) muscles were higher in 2013 than in 2011 and were higher than in Honghu Lake except for Cr.

Huizhou, located in the northeast of the Pearl River Delta, is a typical and famous freshwater aquaculture area for its various fishponds like mulberry-based fishponds, fruit-based fishponds and sugarcane-based fishponds. Table 3 also shows the trace element concentrations in cultured fish muscles of typical areas in Asia, like Korea (in East Asia), Bangladesh (in South Asia), and Malaysia (in Southeast Asia). The concentrations of Cr, Cu, Zn in cultured fish muscles around Honghu Lake were higher than in the other study areas, and the concentration of Pb were also higher than the other areas except Bangladesh.

Table 3 also shows the trace element concentrations of wild and cultured fish from the same areas like Korea and Aegean Sea. The concentrations of Cr and Zn were higher in Korea-cultured fish, and the concentration of Cu was higher in wild fish. The concentrations of Cd, Cr, Cu, Pb, and Zn were slightly higher in Aegean Sea wild fish than in cultured fish. Combining the conclusion of Honghu Lake, living habits (cultured or wild) did not show an obvious influence on trace element distribution in fish muscles. In order to conduct further studies on the factors that influence heavy metal distributions in fish, edible tissues (including the bladder, liver and skin) and some other high enriched organs (including gills, scales and intestines) have been studied.

### 3.2. Trace Element Distributions in Different Tissues of Cultured and Wild Fish

**Figure 1:**
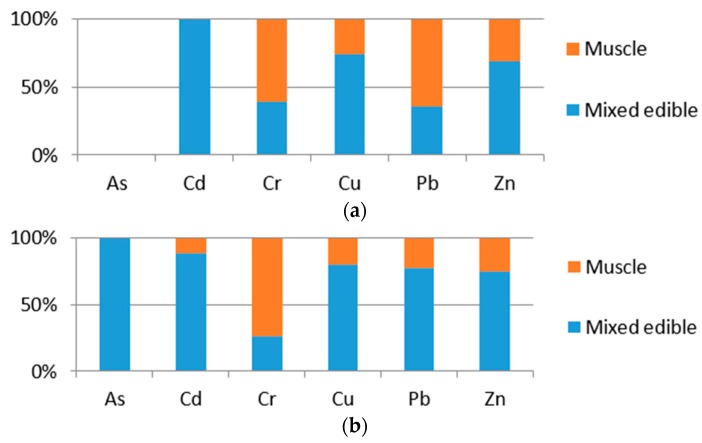
Heavy metals and metalloid concentration (mg/kg wet wt) distribution proportion in muscle and mixed edible tissues of cultured fish (**a**) and wild fish (**b**).

Figure 1a illustrates the proportion of heavy metal distributions in cultured fish (Grass carp and Crucian carp) muscle and mixed edible tissues (bladder, liver, muscle and skin). Figure 1b illustrates the proportion of heavy metal distributions in wild fish (Grass carp and Crucian carp) muscle and mixed edible tissues (bladder, liver, muscle and skin).

With respect to the distribution comparison in fish muscle and mixed fish edible tissues, As was not found in cultured fish muscle or in mixed edible tissues. The concentration proportions of Cd, Cu and Zn in mixed edible tissues were obviously higher than in muscles of both cultured and wild fish. On the contrary, the concentration proportion of Cr was much higher in muscles than in mixed edible tissues of both cultured and wild fish. The concentration proportion of Pb in cultured fish was much higher in muscles than in mixed edible tissues, and in wild fish, was higher in mixed edible tissues. Thus, we can deduce that organs like the liver, bladder, intestine and gill were more likely have higher trace element accumulation abilities in both cultured and wild fish, except for Cr.

**Figure 2:**
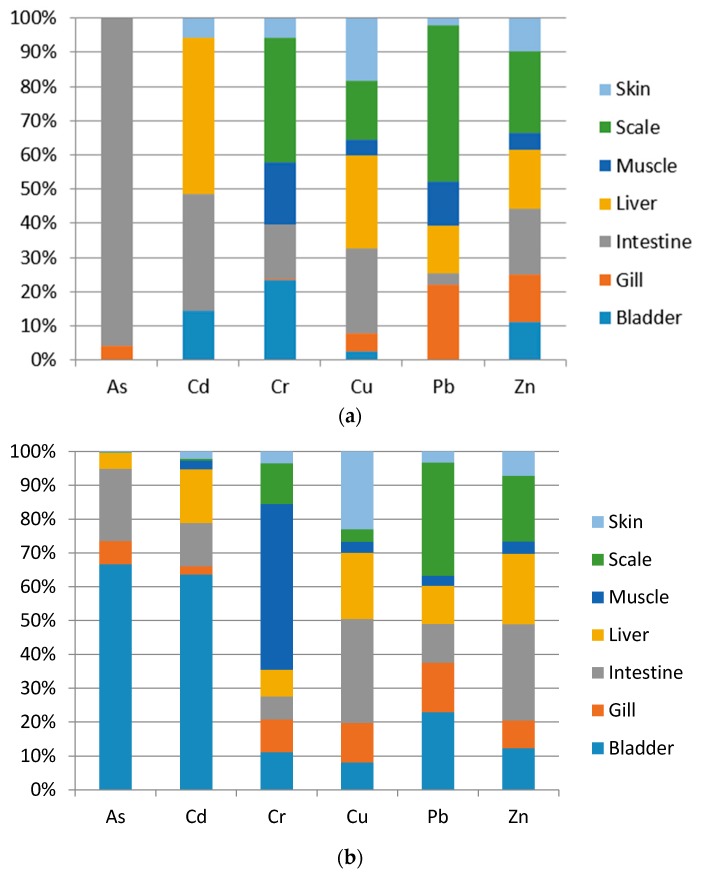
Heavy metals and metalloid concentration (mg/kg wet wt) distribution proportion in different tissues of cultured fish (**a**) and wild fish (**b**).

Figure 2a,b compare the heavy metal distributions in different tissues of cultured (Grass carp and Crucian carp) and wild fish (Grass carp and Crucian carp).

With respect to the distribution comparison among different fish tissues, scales (MPI = 3.342) and liver (MPI = 1.276) were regarded as the main accumulation tissues for cultured fish. As could only be detected in cultured fish intestine and gill, Cd and Cu accumulated mainly in cultured fish intestine and liver, Pb and Cr accumulated mainly in scales, and Zn accumulated evenly in scales, intestines, liver and gills. For wild fish, the bladder (MPI = 0.640) and intestine (MPI = 0.477) were regarded as the main accumulation tissues for wild fish. As and Cd accumulated mainly in wild fish bladder, Cr accumulated mainly in wild fish muscle, Cu and Zn accumulated mainly in intestine and liver, and Pb accumulated mainly in scales and the bladder. To summarize, in both cultured and wild fish, Cu accumulated mainly in the intestine and liver, and Pb accumulated in the scales. This conclusion shows consistency with the results of previous studies [60,61]. Fish can accumulate trace elements through ion exchange with the aquatic environment (like water and sediment) and food ingestion (like water grass, plankton, and feed) [62,63]. The main accumulation tissues for cultured fish were the scales and liver, and for wild fish, the bladder and intestine. This conclusion may reveal that the heavy metal concentration in an aquatic environment may influence cultured fish more than wild fish, and the heavy metal concentration in fish food more than in wild fish. To demonstrate this conclusion, aquaculture water and sediment, lake water and sediment, plankton, typical grass and artificial feed can be included in the investigation subjects for further studies of trace element distributions.

### 3.3. Comparison of Health Risk Assessment for Cultured and Wild Fish

Residual heavy metals and metalloids in fish will be amplified and enriched by the food chain, posing a serious threat to human health and the ecological environment. Thus, it is necessary to assess the corresponding health risk brought about by cultured and wild fish consumption. The daily fish muscle consumption of inhabitants in Hubei province is 54.33 g/day [46]. Table 4 shows the calculation results of target hazard quotients (THQ), carcinogenic risk (CR) and estimated weekly intake (EWI) from cultured and wild fish (muscle and mixed edible tissues) consumption. All the calculated results of THQ, CR and EWI were based on the mean heavy metal concentrations of cultured and wild fish (Grass carp and Crucian carp) muscle and mixed edible tissues (bladder, liver, muscle and skin).

For cultured fish, the calculation results of THQ for all trace elements through cultured fish muscle and mixed edible tissue consumption were less than 1, which means there were no significant health risks through cultured fish consumption. The health risk levels of cultured fish muscle calculated by THQ decreased in the following order, Zn > Pb > Cu > Cr. The health risk levels for mixed edible tissues of cultured fish calculated by THQ decreased in the following order, Zn > Cu > Pb > Cd > Cr. The above calculation results of THQ reveal that without considering the nutrient elements (Zn and Cu), Pb (accounts more than 70% of the total target hazard quotient in muscle) was recognized as the major contributor of non-carcinogenic risk to the local inhabitants for both cultured fish muscle and mixed edible tissue consumption. The calculation results of CR for Pb through cultured fish muscle consumption was recognized as acceptable (1.0 × 10^−6^ < 1.47 × 10^−6^ < 1.0 × 10^−4^), and through mixed edible tissues consumption was recognized as negligible (8.30 × 10^−7^ < 1.0 × 10^−6^). The calculation results of EWI for all trace elements through cultured fish muscle and mixed edible tissues were less than the corresponding PTWIs (the limitations of EWI), which means there were no significant health risks through cultured fish consumption. EWI/PTWI values were selected to compare the health risks of different trace metals. The EWI/PTWI values in cultured fish muscles decreased in the following order: Cr > Pb > Zn > Cu. The EWI/PTWI values in mixed edible tissues of cultured fish decreased in the following order, Cr > Zn > Pb > Cd > Cu. The above calculation results of EWI/PTWI revealed that Cr was recognized as the major contributor of non-carcinogenic risk to the local inhabitants for both cultured fish muscle and mixed edible tissue consumption.

For wild fish, the calculation results of THQ for all trace elements through wild fish muscle and mixed edible tissue consumption were less than 1, which means there were no significant health risks through wild fish consumption. The health risk levels of wild fish muscle calculated by THQ decreased in the following order, Zn > Pb > Cu > Cd > Cr. The health risk levels in mixed edible tissues of wild fish calculated by THQ decreased in the following order, Zn > Cd > Pb > Cu > As > Cr. The above calculation results of THQ reveal that without considering the nutrient elements (Zn and Cu), Pb (accounts for nearly 70% of the total target hazard quotient) was recognized as the major contributor of non-carcinogenic risk for wild fish muscle consumption to the local inhabitants. Cd, Pb, Cu and As had nearly equal potential non-carcinogenic risks in mixed edible tissues of wild fish consumption. The calculation results of CR for As and Pb through mixed edible tissue consumption of wild fish were recognized as acceptable (1.0 × 10^−6^ < 1.60 × 10^−5^ < 1.0 × 10^−4^, 1.0 × 10^−6^ < 1.30 × 10^−6^ < 1.0 × 10^−6^), and for Pb through muscle consumption, negligible (3.77 × 10^−7^ < 1.0 × 10^−6^). The above calculated results of CR reveal that As was recognized as the major contributor of carcinogenic risk for local inhabitants through wild fish mixed edible tissue consumption, and Pb was recognized as the major contributor through wild fish muscle consumption. The calculation results of EWI for all trace elements in wild fish muscle and mixed edible tissues were less than the corresponding PTWIs, and that means there were no significant health risks associated with wild fish consumption. The EWI/PTWI values for wild fish muscles decreased in the following order, Cr > Zn > Pb > Cd > Cu. The EWI/PTWI values for mixed edible tissues of wild fish decreased in the following order, Cr > Pb > Zn > Cd > As > Cu. The above calculation results of EWI/PTWI reveal that Cr was recognized as the major contributor of non-carcinogenic risk to the local inhabitants for both wild fish muscle and mixed edible tissue consumption.

Overall, for both cultured and wild fish, Pb was recognized as the major contributor for non-carcinogenic risk and carcinogenic risk assessment based on the calculation results of THQ and CR, and Cr was recognized as the major contributor for non-carcinogenic risk based on the calculation results of EWI. Therefore, Pb and Cr were selected as the representative trace elements to compare the potential health risk between cultured fish and wild fish, and between muscle consumption and mixed edible tissue consumption.

**Figure 3:**
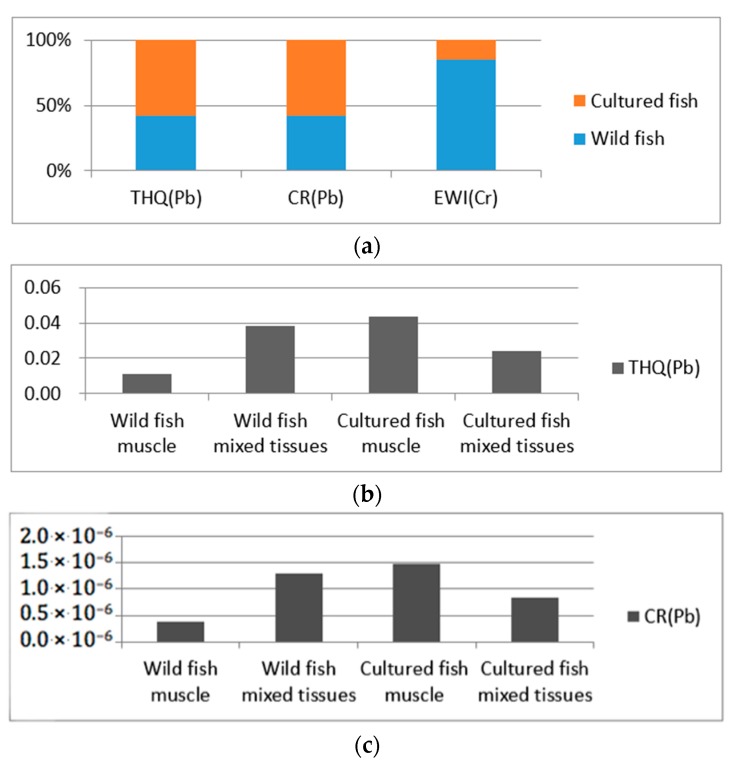

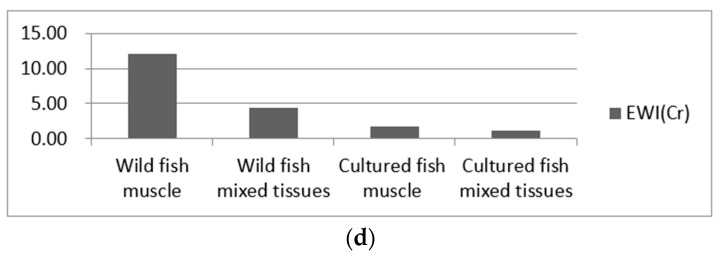
Calculation results of health risks of wild and cultured fish (**a**) through fish consumption based on target hazard quotients (**b**), carcinogenic risk (**c**) and estimated weekly intake (**d**).

Figure 3a shows that cultured fish was associated with more health risks than wild fish based on the calculation results of THQ and CR, and wild fish was associated with more risk than cultured fish based on the calculation results of EWI. Figure 3b–d shows that in cultured fish, muscle consumption had higher potential health risk than mixed edible tissues, based on the calculation results of THQ, CR and EWI. However, in wild fish, mixed edible tissues had higher potential health risk than muscle.

**Figure 4:**
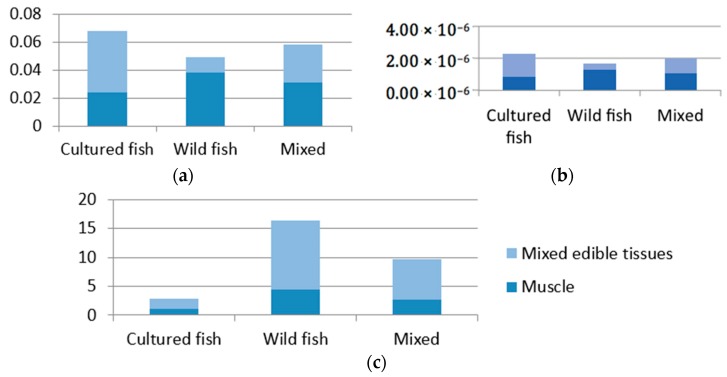
Calculation results of health risks through cultured, wild and mixed (wild:cultured = 1:1) fish muscle and mixed edible tissue consumption based on THQ (**a**), CR (**b**) and EWI (**c**).

In order to explore the method for minimizing health risks through fish consumption, this study calculated the health risk associated with mixed fish (wild:cultured =1:1) consumption to balance the risks calculated by three different models. The total fish ingestion rate remained 54.33 g/day. The calculation results of consuming cultured fish mixed with wild fish compared with consuming cultured fish or wild fish separately are shown in Figure 4a–c.

Figure 4 shows that, for fish muscle, consuming cultured fish separately brought relatively lower health risks than wild and mixed fish through fish consumption. When the study objectives involved all edible fish tissues besides muscle, consuming cultured fish mixed with wild fish brought relatively lower health risks than consuming wild and mixed fish separately, considering the comprehensive assessment results based on THQ, CR and EWI.

### 3.4. Comparison of the Maximum Allowable Fish Daily Consumption Limit for Cultured and Wild Fish

Table 5 shows the calculation results of the maximum allowable fish daily consumption (${\mathrm{FIR}}_{\lim}$) based on target hazard quotients (THQ), carcinogenic risk (CR) and estimated weekly intake (EWI).

Based on the health risk calculation model of THQ, for fish muscle, people should consume less than 1018 g/day cultured fish muscle or 1183 g/day wild fish muscle from Honghu Lake to assure their health. For mixed edible fish tissues, people were suggested to consume less than 425 g/day cultured fish mixed tissues or 396 g/day wild fish mixed tissues to assure their health. Based on the health risk calculation model of CR, for fish muscle, people should consume less than 3684 g/day cultured fish muscle or 14,396 g/day wild fish muscle to assure their health. For mixed edible fish tissues, people should consume less than 6550 g/day cultured fish mixed tissues or 340 g/day wild fish mixed tissues to assure their health. Based on the health risk calculation model of EWI, for fish muscle, people should consume less than 465 g/day cultured fish muscle or 68 g/day wild fish muscle to assure their health. For mixed edible fish tissues, people should consume less than 721 g/day cultured fish mixed tissues or 186 g/day wild fish mixed tissues to assure their health. Considering the comprehensive calculated results based on THQ, CR and EWI, people should consume no more than 465 g/day cultured fish muscle, 68 g/day wild fish muscle, 452 g/day mixed cultured fish edible tissues or 186 g/day mixed wild fish edible tissues to assure their health to the greatest extent.

## 4. Conclusions

This study provides valuable information concerning comparison of toxic metal distributions, health risk assessment and fish consumption limitation for cultured and wild fish around Honghu area. In general, all detected trace elements in studied fish were within the corresponding standards (GB2762-2012 and NY5073-2006). High levels of Cr accumulated in wild fish muscles (1.947 mg/kg), and relatively high levels of Pb accumulated in cultured fish muscles (0.2052 mg/kg). Gills, intestines, the liver and scales were the main heavy metal accumulation organs for cultured fish, compared with the bladder, intestines, liver and scales for wild fish. Pb and Cr were recognized as the major contributors of non-carcinogenic risk to the local inhabitants for both wild and cultured fish consumption. For local government, the Cr concentration in Honghu Lake should be regularly monitored because the corresponding health risk calculated by EWI was almost close to the limitation (EWI_Cr_/PTWI_Cr_ = 80.13%). Cultured fish had more health risks than wild fish based on the calculation results of THQ and CR; in contrast, wild fish had the opposite results based on the calculation results of EWI. Thus, for local inhabitants, consuming muscle of cultured fish separately had relatively lower potential health risks, and when the consumption objectives were mixed edible fish tissues, a ratio of 1:1 for cultured fish mixed with wild fish was recommended. In addition, people should consume no more than 465 g/day of cultured fish muscle, 68 g/day of wild fish muscle, 452 g/day of mixed cultured fish edible tissues or 186 g/day of mixed wild fish edible tissues from Honghu Lake to assure their health to the greatest extent.

## Figures and Tables

**Table 1:** Single and multi factor pollution indexes of trace elements in cultured fish muscle.

Fish Species	P_i_						MPI
	As	Cd	Cr	Cu	Pb	Zn	
Crucian carp	0.0000	0.0000	0.0957	0.0136	0.2518	0.2788	0.0977
Grass carp	0.0000	0.0000	0.1884	0.0204	0.5688	0.4786	0.1798
Carp	0.0000	0.0260	0.0513	0.0114	0.1900	0.6478	0.0921
Catfish	0.0000	0.0720	0.0705	0.0133	0.9316	0.2976	0.1269

MPI: multi factor pollution index.

**Table 2:** Trace element concentrations in cultured and wild fish muscles (mg/kg).

Fish Species		As	Cd	Cr	Cu	Pb	Zn
Cultured	Crucian carp	0.0000	0.0000	0.1913	0.6797	0.1259	13.94
	Grass carp	0.0000	0.0000	0.3767	1.018	0.2844	23.93
	Mean	0.0000	0.0000	0.2840	0.8489	0.2052	18.94
Wild	Crucian carp	0.0000	0.0087	3.357	0.3829	0.0938	15.31
	Grass carp	0.0000	0.0032	0.5369	0.4502	0.0111	17.29
	Mean	0.0000	0.0060	1.947	0.4166	0.0525	16.30
LOD *		0.001	0.001	0.001	0.02	0.01	0.1
Chinese standard [51,52]		0.1	0.1	2.0	50	0.5	50

* LOD: Limit of Detection.

**Table 3:** Trace element concentrations in fish muscles of typical areas (mg/kg; wet wt).

Species	Nation	Lake/River	As	Cd	Cr	Cu	Pb	Zn	Reference
Wild	China	Honghu Lake	0.0000	0.0060	1.947	0.4166	0.0525	16.30	This study
Cultured			0.0000	0.0000	0.2840	0.8489	0.2052	18.94	
Wild	China	Poyang Lake	0.0275	0.0045	0.2510	0.4165	0.0505	7.785	[30]
		Taihu Lake	0.6300	0.4740	1.1820	1.1160	5.8140	81.3	[53]
		Yangtze River	0.0145	0.1120	0.1750	0.9800	0.5700	6.800	[54]
			0.5640	0.1605	0.6255	1.3875	1.4865	35.57	[53]
Cultured	China	Huizhou	-	-	0.1592	0.1520	0.0606	5.140	[55]
	Korea	-	-	0.033	-	-	0.069	-	[56]
	Bangladesh	-	0.332	0.017	0.193	-	0.593	-	[57]
	Malaysia	-	0.7967	0.0297	-	0.21	0.0137	3.7	[58]
Wild	Korea	-	-	0.0000	0.10	1.15	0.0000	6.18	[59]
Cultured		-	-	0.0000	0.51	0.96	0.0000	6.92	
Wild	Aegean Sea	-	-	0.11	0.37	1.31	0.48	14.38	[28]
Cultured		-	-	0.05	0.33	0.56	0.45	7.53	

**Table 4:** Calculation results of target hazard quotients (THQ), carcinogenic risk (CR) and estimated weekly intake (EWI) from cultured and wild fish consumption.

Trace Elements			As	Cd	Cr	Cu	Pb	Zn
Cultured	Muscle	Mean concentration (mg/kg)	0	0	0.284	0.8489	0.2051	18.94
	Mixed tissues		0	0.0124	0.1831	2.430	0.1154	42.63
Wild	Muscle		0	0.0060	1.947	0.4166	0.0525	16.30
	Mixed tissues		0.0126	0.0475	0.711	1.706	0.1809	48.68
R_FD_^1^ (mg/kg/day)			0.0003	0.001	1.5	0.04	0.004	0.3
Cultured	Muscle	THQ	0	0	0.0002	0.0179	0.0434	0.0534
	Mixed tissues		0	0.0105	0.0001	0.0514	0.0244	0.1202
Wild	Muscle		0	0.0050	0.0011	0.0088	0.0111	0.0459
	Mixed tissues		0.0355	0.0402	0.0004	0.0361	0.0382	0.1372
Cultured	Muscle	CR	0	-	-	-	1.47 × 10^−6^	-
	Mixed tissues		0	-	-	-	8.30 × 10^−7^	-
Wild	Muscle		0	-	-	-	3.77 × 10^−7^	-
	Mixed tissues		1.60 × 10^−5^	-	-	-	1.30 × 10^−6^	-
PTWI ^2^ (µg/kg)			15	7	15	3500	25	7000
Cultured	Muscle	EWI (µg/kg)	0	0	1.754	5.241	1.267	117
	Mixed tissues		0	0.0768	1.131	15.01	0.7124	263
Wild	Muscle		0	0.0368	12.02	2.572	0.3238	101
	Mixed tissues		0.0778	0.2933	4.390	10.53	1.117	301
Cultured	Muscle	EWI/PTWI	0	0	11.69%	0.15%	5.07%	1.67%
	Mixed tissues		0	1.10%	7.54%	0.43%	2.85%	3.76%
Wild	Muscle		0	0.53%	80.13%	0.07%	1.30%	1.44%
	Mixed tissues		0.52%	4.19%	29.26%	0.30%	4.47%	4.29%

^1^ R_FD_ values referenced from USEPA, 2010 [39]; ^2^ PTWI values referenced from FAO, 2006 [64].

**Table 5:** Calculation results of food daily consumption (F_IR_) limitation based on THQ, CR and EWI.

Food Ingestion Rate (F_IR_) Limitation (g/day)		As	Cd	Cr	Cu	Pb	Zn
Based on target hazard quotients (THQ)							
Cultured fish	Muscle	/	/	339,296	3027	1253	1018
	Mixed tissues	/	5162	526,225	1057	2227	452
Wild fish	Muscle	/	10,707	49,497	6168	4894	1183
	Mixed tissues	1529	1352	135,527	1506	1420	396
Based on carcinogenic risk (CR)							
Cultured fish	Muscle	/	/	/	/	3684	/
	Mixed tissues	/	/	/	/	6550	/
Wild fish	Muscle	/	/	/	/	14,396	/
	Mixed tissues	340	/	/	/	4178	/
Based on estimated weekly intake (EWI)							
Cultured fish	Muscle	/	/	465	36,284	1072	3253
	Mixed tissues	/	4950	721	12,673	1907	1445
Wild fish	Muscle	/	10,267	68	73,932	4190	3780
	Mixed tissues	10,475	1297	186	18,052	1216	1265

## Supplementary Materials

The following are available online at http://www.mdpi.com/1660-4601/15/2/334/s1; Figure S1: Map of cultured fishes sampling fishponds (red points S1-6) around Honghu Lake, Table S1: Species, feeding habits, lengths (Mean ± SD) and weights (Mean ± SD) for cultured fishes, Table S2: Species, feeding habits, lengths (Mean ± SD) and weights (Mean ± SD) for wild fishes.
