# Supplementary material for: Comparison of Toxic Metal Distribution Characteristics and Health Risk between Cultured and Wild Fish Captured from Honghu City, China
Source: Int J Environ Res Public Health. 2018 Feb 14;15(2):334. doi: 10.3390/ijerph15020334 (PMC5858403; doi:10.3390/ijerph15020334)
Supplement: Supplementary file 1 [file ijerph-15-00334-s001.pdf]

Supplementary Material

Table S1. Species, feeding habits, lengths (Mean ± SD) and weights (Mean ± SD) for cultured fishes

Common Name	Scientific Name	Feeding Habits	n	Length (cm)	Weight (g)
Catfish	Silurus asotus	Carnivorous	8	40.15±1.95	615±22
Carp	Cyprinus carpio	Omnivorous	8	36.35±2.05	614±22
Crucian carp	Carassius auratus	Omnivorous	8	30.80±2.10	413±24
Grass carp	Ctenopharyngodon idellus	Herbivorous	8	45.70±2.10	1104±106

Table S2. Species, feeding habits, lengths (Mean ± SD) and weights (Mean ± SD) for wild fishes

Common Name	Scientific Name	Feeding Habits	n	Length (cm)	Weight (g)
Crucian carp	Carassius auratus	Omnivorous	8	20.50±2.10	175±14
Grass carp	Ctenopharyngodon idellus	Herbivorous	8	35.55±3.35	576±26

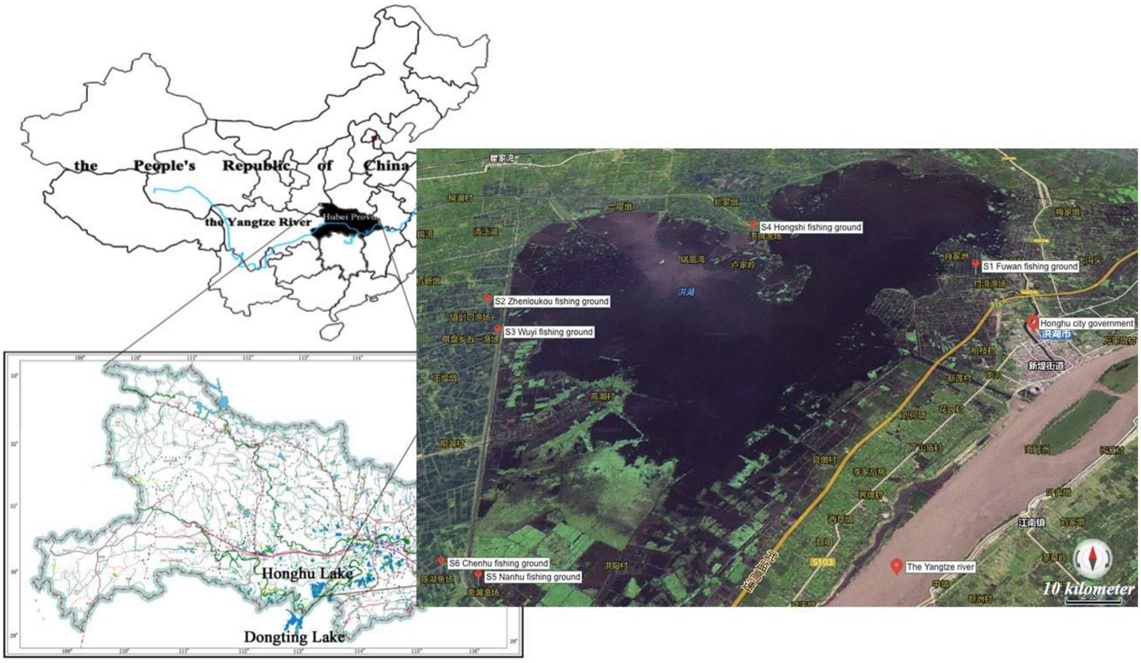

Figure S1. Map of cultured fishes sampling fishponds (red points S1-6) around Honghu Lake
